# Evaluation of Fentanyl Exposure Effects on Butyrylcholinesterase Activity as a Tool for Future On-Site Detection Methods

**DOI:** 10.1021/acsomega.4c06655

**Published:** 2024-09-14

**Authors:** Vrunda Rania, Ashley Newland, Lenka Halámková, Václav Trojan, Radovan Hřib, Jan Halámek

**Affiliations:** † Department of Environmental Toxicology, The Institute for Forensic Science, Texas Tech University, 1207 Gilbert Drive, Lubbock, Texas 79416, United States; ‡ Cannabis Facility, International Clinical Research Centre, St. Anne’s University Hospital, Brno 60200, Czech Republic; § Department of Natural Drugs, Faculty of Pharmacy, Masaryk University, Palackého 1946/1, 61200 Brno, Czech Republic; ∥ Cannabis Facility, International Clinical Research Centre, St. Anne’s University Hospital, Brno 60200, Czech Republic; ⊥ Center for Pain Management, Department of Anesthesiology and Intensive Care, St. Anne’s University Hospital, Brno 60200, Czech Republic

## Abstract

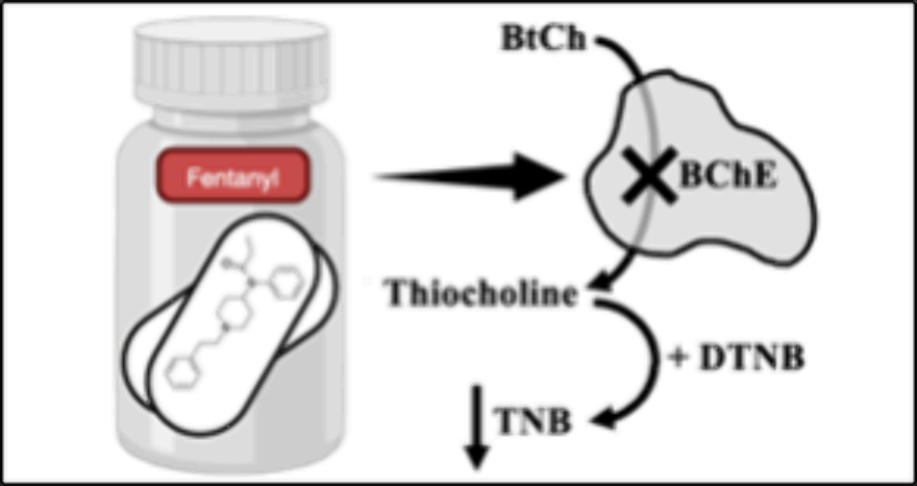

The prominence of fentanyl and fentanyl analogues or Fentanyl Related Substances (FRS) has driven a nationwide crisis of opioid overdoses, which significantly presents an issue for public health and safety. Originally developed for medical purposes, fentanyl and FRS have become critical contributors to opioid overdose deaths due to their distribution, availability, and potency. This study examined toxicodynamic properties between butyrylcholinesterase (BChE) and fentanyl analogues via Ellman’s assay. The enzymatic function of BChE was significantly inhibited by each of the 5 fentanyl analogues tested, which indicates the potential for utilization of this interaction. This reaction can be immobilized for a portable, single-use kit to detect FRS directly from any surface on-site. This would immensely benefit society by reducing the frequency of exposure and overdoses by providing additional safety measures to law enforcement and first responders.

## Introduction

Fentanyl is a synthetic opioid developed by Janssen in the 1960s as an intravenous anesthetic and gained FDA approval for human medical use in 1968.^1,2^ It is also used as an anesthetic in veterinary medicine for large animals.^2,3^ The U.S. Drug Enforcement Agency (DEA) classifies fentanyl and fentanyl analogues or Fentanyl-Related Substances (FRS) as Schedule II substances based on high abuse potential.^4^ The DEA states that fentanyl and FRS are considered dangerous since it is known to be up to 10,000 times more potent than morphine dependent on the specific analogue.^2,4^ Trafficking, distribution, and consumption of fentanyl and FRS have contributed to the nationwide opioid epidemic.^1,2^ In 2017, the Centers for Disease Control and Prevention (CDC) declared a public health emergency that arose from a 5-fold increase in opioid overdoses from 2012 to 2016.^5^ Fentanyl was determined to be associated with 57,834 deaths in 2020 and 71,238 deaths in 2021.^6^ According to the CDC, fentanyl and FRS were involved in 66% of all drug overdose fatalities in 2021.^7^

The precursor for fentanyl is predominantly sourced from China and imported from Mexico where it is produced and distributed across the U.S. from Mexico’s borders.^2^ In 2023, the U.S. Border Patrol and Office of Field Operations collaboratively seized 27,000 lbs of fentanyl, which is a 40% increase since 2021.^8^ Drug traffickers mix fentanyl and FRS with other illicit drugs such as cocaine, heroin, and methamphetamine to increase the potency and financial profit from products. This practice leads to users and victims unknowingly ingesting fentanyl and FRS.^9,10^ In 2023, the DEA seized 77 million fentanyl pills along with 12,000 lbs of fentanyl powder.^11^ Fentanyl is not visually distinguishable from other illicit drugs in powder form, but fentanyl and FRS are far more potent than other illicit substances.^12^ Therefore, a rapid detection method is necessary for law enforcement and first responders to determine the specific substance(s) directly handled at the scene. Knowledge of fentanyl surface contamination is a key factor in minimizing accidental overdose risk.^13^

The Lateral Flow Immunoassay (LFI)-fentanyl test strips (FTS) and Enzyme-linked Immunosorbent Assay (ELISA) tests are currently used for fentanyl detection.^14^ FTS require a 2–5 min waiting period that is crucial to the safety of first responders, law enforcement, and other individuals who may have been exposed to fentanyl or FRS as overdoses can occur within this time frame. The presence of additional illicit substances and heterogenicity within the sample can affect the FTS results. Although FTS can detect nanogram levels of fentanyl and FRS with a minimum of 96% accuracy in numerous forms (pills, powder, injectables), it is useful to evaluate additional techniques. It is recommended that a minimum of 10 mg or 5 mL, dependent on the form obtained, is required for FTS to accurately and precisely provide positive or negative results.^14^

Other technological advancements have allowed law enforcement to detect fentanyl via hand-held Fourier Transform Infrared spectrometers and Raman spectrometers. Using these hand-held devices provides a safe, efficient, and intuitive method for individuals to analyze samples accurately without contact.^15^ Routine sample analyses with spectroscopic devices may not be available for agencies that do not have the required funding since the hand-held FT-IR and Raman analyzers cost thousands of dollars. Given the cost efficiency, accuracy, and precision, FTS technology can be explored and enhanced to require a smaller sample volume and decreased response time while still providing accuracy at ng levels. LFI-FTS is deemed to be a useful detection tool, but knowledge of biochemical interactions may provide another mode for FTS analysis.

Prior studies concur there is an interaction between butyrylcholinesterase (BChE) and choline-based esters such as organophosphates, carbamate, cocaine, and heroin.^16−18^ It is suggested that BChE hydrolyzes methyl ester and benzoyl ester sites to form inactive metabolites.^16,18,19^ Heroin is also metabolized by BChE to produce morphine.^16^ In a previous study by Taghizadehghalehjoughi et al., the effect of fentanyl and remifentanil on neuron damage and oxidative stress during neurotoxicity induction was evaluated using whole-cell specimens. It was suggested that BChE activity decreased while acetylcholinesterase (AChE) activity remained relatively constant providing insight into the concept that fentanyl specifically interacts with BChE.^20^ However, the interaction between BChE and fentanyl has remained unknown due to the study of complex specimens, which may contribute to the conclusion that BChE inactivity is a direct result of fentanyl exposure as opposed to unknown mechanisms influencing decreased enzymatic activity.

This research aims to provide proof of concept that an interaction between BChE and fentanyl analogues occurs by direct molecule exposure in solution versus evaluation of complex systems to fill the gap in existing research. This study assessed BChE function from exposure to 5 different fentanyl analogues at variable concentrations with Ellman’s Assay.^21^ Results demonstrate inhibition of BChE from all fentanyl analogues tested in concentrations from 1 × 10^–7^ M to 1 × 10^–12^ M. These fundamental findings can lead to developments in technology that can detect FRS in addition to current techniques. The Ellman’s assay implemented onto a single-use portable kit-like device would immensely impact law enforcement, first responders, and the general public by providing an accurate technique for the evaluation of surface contamination and illicit substances on-site.

## Experimental Section

### Materials

The following enzymes and reagents utilized during this study were purchased from Millipore Sigma (Burlington, MA): butyrylcholinesterase from equine serum (BChE; EC 3.1.1.8), 5,5-dithiobis(2-nitrobenzoic acid) (DTNB), s-butyrylthiocholine iodide (butyrylthiocholine or BtCh), potassium phosphate monobasic, potassium phosphate dibasic, and 100% methanol. The fentanyl analogues were chosen based on availability and purchased from Cayman Chemical Company (Ann Arbor, MI): benzyl acrylfentanyl (hydrochloride), benzyl carfentanil (hydrochloride), *N*-benzyl furanyl norfentanyl (hydrochloride), norsufentanil, and desopropionyl meta-methyl fentanyl. A Barnstead Nano-Pure purification system from Thermo-Scientific (Waltham, MA) was used to obtain ultrapure (18.2 MΩ·cm) water. All analyses were performed at 37 °C using the BioTek Synergy H1 microplate reader from Agilent with 96-well microtiter polystyrene plates purchased from Avantor-VWR (Radnor, PA).

### Methods

#### Optimization of Ellman’s Assay

Before BChE exposure to all fentanyl analogues, optimization of Ellman’s assay was completed with the following reaction components: 50 mM potassium phosphate buffer, pH 7.4, 25 mU BChE, 500 μM DTNB, and variable BtCh concentrations at 10 μM, 25 μM, 50 μM, 75 μM, 100 μM, 125 μM, and 150 μM. Each concentration was measured in triplicate at 405 nm for 600s at 37 °C using the BioTek Synergy H1 microplate reader.

#### Ellman’s Assay with Fentanyl Analogues

Following protocol optimization, the components were added to a 96-well microtiter polystyrene plate in the following order: 50 mM potassium phosphate buffer, pH 7.4, 25 mU BChE, 1 × 10^–7^ to 1 × 10^–12^ M fentanyl analogue, 500 μM DTNB, and 100 μM BtCh. Each FRS concentration was measured in triplicate at 405 nm for 600s at 37 °C using the BioTek Synergy H1 microplate reader.

#### Statistical Analysis

Excel was used to determine the change in absorbance, triplicate response averages, standard deviations, and percent inhibition values. Enzyme inhibition was calculated by (Abs_blank_ – Abs_sample_)/Abs_blank_ × 100. All graphs were generated using OriginPro 9.1.

## Results

### Optimization of Ellman’s Assay

**Figure 1:**
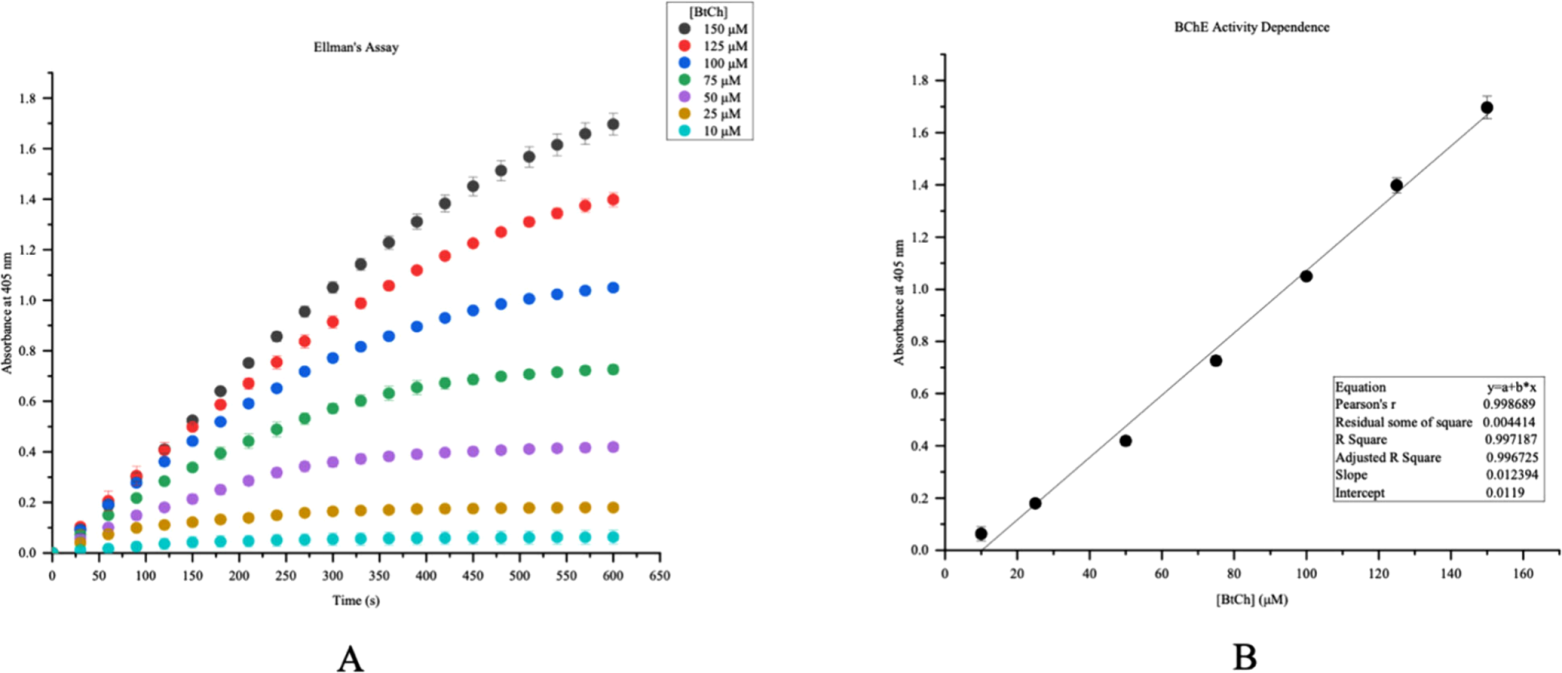
Ellman’s Assay shows a calibration curve generated from variable BtCh concentrations with constant substrate concentrations. Absorbance values at 405 nm range from approximately 0.1–1.7 with 10 μM BtCh producing the lowest response (0.1) and 150 μM BtCh producing the highest response (1.7) at 600s (A). BChE Activity Dependence shows a strong linear correlation of 10 μM–150 μM BtCh with 25 mU BChE (B).

Concentrations of BtCh were measured at 10 μM, 25 μM, 50 μM, 75 μM, 100 μM, 125 μM, and 150 μM to assess optimal concentration performance, which was determined to be 100 μM based on ideal signal responses shown in Figure 1A. Determination of the 4:1 substrate-to-enzyme ratio was essential to yield the most accurate comparison between control samples and fentanyl-treated samples. It should be noted that control sample values are within the optimal 1–2 absorbance range for all experiments and demonstrate maximum efficacy of BtCh hydrolysis via BChE.^22^

The calibration curve for Ellman’s assay depicted in Figure 1B shows the linear correlation between variable concentrations of BtCh with 25 mU BChE at 405 nm for 600s. The *R*^2^ value was 0.997, and the Pearson’s *R* value was 0.999. These values serve to highlight a strong substrate concentration dependence of BtCh hydrolysis by 25 mU of BChE.

### Determination of BChE Activity with Fentanyl Analogues

**Figure 2:**
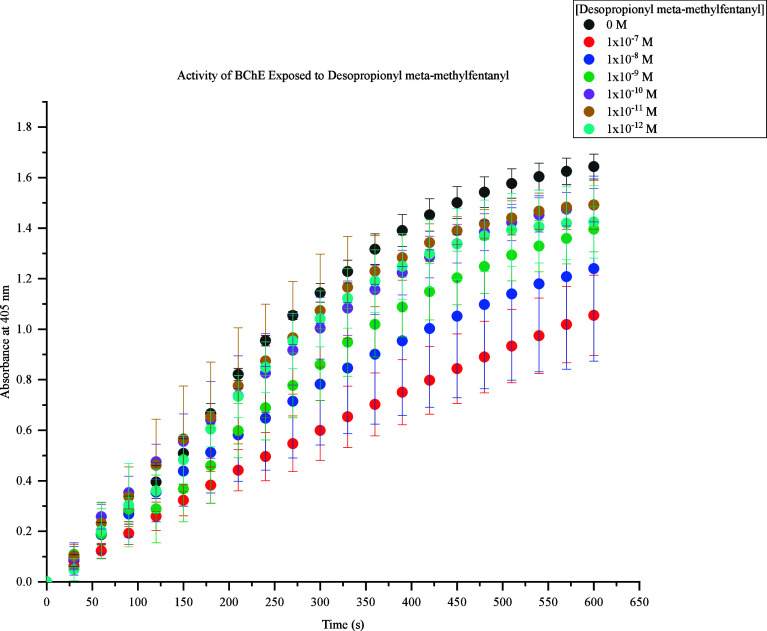
Activity of BChE Exposed to Desopropionyl meta-methylfentanyl. Response values at 405 nm range from approximately 0.6–1.6 at 600s. The highest concentration (1 × 10^–7^ M) produced the lowest response (0.6), and the lowest concentrations (1 × 10^–10^ M to 1 × 10^–12^ M) produced the highest response (1.4). At 600s, 1 × 10^–10^ M to 1 × 10^–12^ M caused a decrease in BChE activity by 0.1 compared to the control samples.

The first fentanyl analogue assessed was desopropionyl meta-methylfentanyl. This FRS demonstrated the inhibition of BChE activity at all concentrations tested, as shown in Figure 2. Significant BChE inhibition was observed at higher concentrations of 1 × 10^–7^ M to 1 × 10^–9^ M. Lower concentrations of 1 × 10^–10^ M to 1 × 10^–12^ M produced indistinguishable values due to lack of inhibitory difference. Significant BChE inhibition was further expressed as concentrations of desopropionyl meta-methyl fentanyl increased.

**Figure 3:**
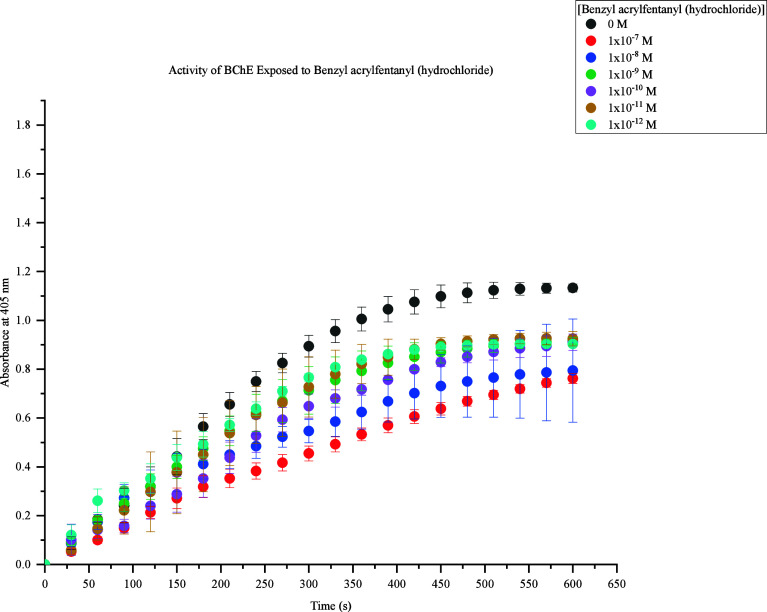
Activity of BChE Exposed to Benzyl acrylfentanyl (hydrochloride). Response values at 405 nm range from approximately 0.7–1.1 at 600s. The highest concentration (1 × 10^–7^ M) produced the lowest response (0.7), and the lowest concentrations (1 × 10^–10^ M to 1 × 10^–12^ M) produced the highest response (0.9). At 600s, 1 × 10^–9^ M to 1 × 10^–12^ M caused a decrease in BChE activity by 0.2 compared to the control samples.

The second fentanyl analogue acquired was benzyl acrylfentanyl (hydrochloride). This FRS demonstrated inhibition of BChE activity at all concentrations tested, as shown in Figure 3. The lowest concentrations of 1 × 10^–10^ M to 1 × 10^–12^ M strongly inhibited BChE. It was observed that 1 × 10^–10^ M exhibited greater inhibition initially as compared to 1 × 10^–9^ M. Significant BChE inhibition was further expressed as concentrations of benzyl acrylfentanyl (hydrochloride) increased.

**Figure 4:**
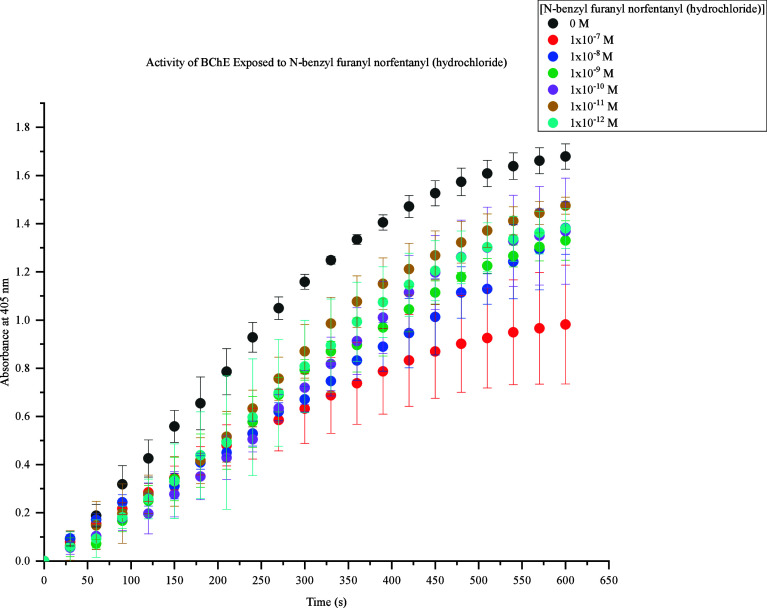
Activity of BChE Exposed to *N*-benzyl furanyl norfentanyl (hydrochloride). Response values at 405 nm range from approximately 0.9–1.7 at 600s. The highest concentration (1 × 10^–7^ M) produced the lowest response (0.9), and the lowest concentrations (1 × 10^–8^ M to 1 × 10^–12^ M) produced the highest responses (1.2–1.4). At 600s, 1 × 10^–8^ M to 1 × 10^–12^ M caused a decrease in BChE activity by 0.2–0.4 compared to the control samples.

The third fentanyl analogue assessed was *n*-benzyl furanyl norfentanyl (hydrochloride). This FRS demonstrated inhibition of BChE activity at all concentrations tested, as shown in Figure 4. Indistinguishability was observed between 1 × 10^–8^ M and 1 × 10^–12^ M at 600s due to lack of inhibitory difference when these concentrations were evaluated at this interval. However, 1 × 10^–10^ M up to 500s does show a lower response compared to 1 × 10^–9^ M, 1 × 10^–11^, and 1 × 10^–12^, but this response still does not provide distinguishability. Significant BChE inhibition was further expressed with 1 × 10^–7^ M *n*-benzyl furanyl norfentanyl (hydrochloride).

**Figure 5:**
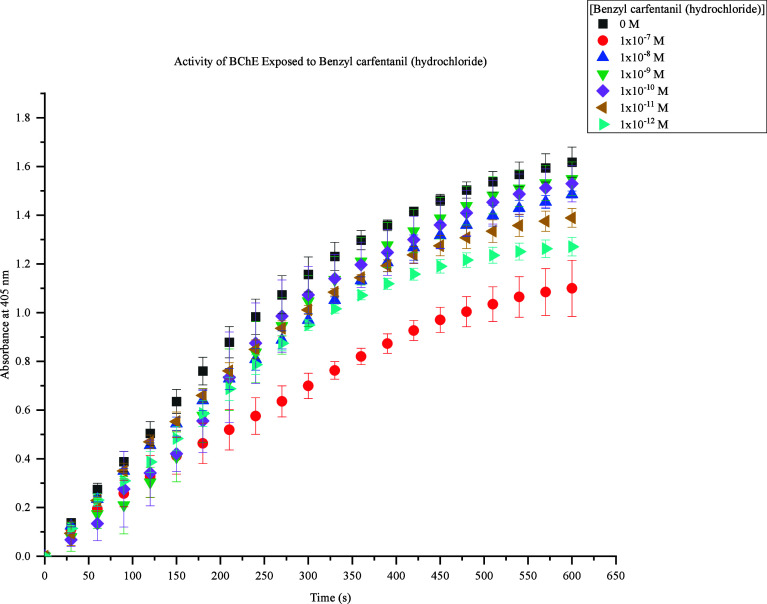
Activity of BChE Exposed to Benzyl carfentanil (hydrochloride). Response values at 405 nm range from approximately 1.0–1.6 at 600s. The highest concentration (1 × 10^–7^ M) produced the lowest response (1.0), and the lowest concentrations (1 × 10^–8^ M to 1 × 10^–12^ M) produced the highest responses (1.2–1.5). At 600s, 1 × 10^–8^ M to 1 × 10^–12^ M caused a decrease in BChE activity by 0.1–0.4 compared to the control samples.

The fourth fentanyl analogue assessed was benzyl carfentanil (hydrochloride). This FRS demonstrated insignificant inhibition of BChE activity between 1 × 10^–8^ M to 1 × 10^–12^ M shown in Figure 5. Distinguishability was observed with 1 × 10^–7^ M, but concentration dependence cannot be observed at lower levels. However, there is distinguishability between 1 × 10^–8^ M to 1 × 10^–12^ M and the control sample, which suggests there is an effect on BChE activity when exposed to benzyl carfentanil (hydrochloride).

**Figure 6:**
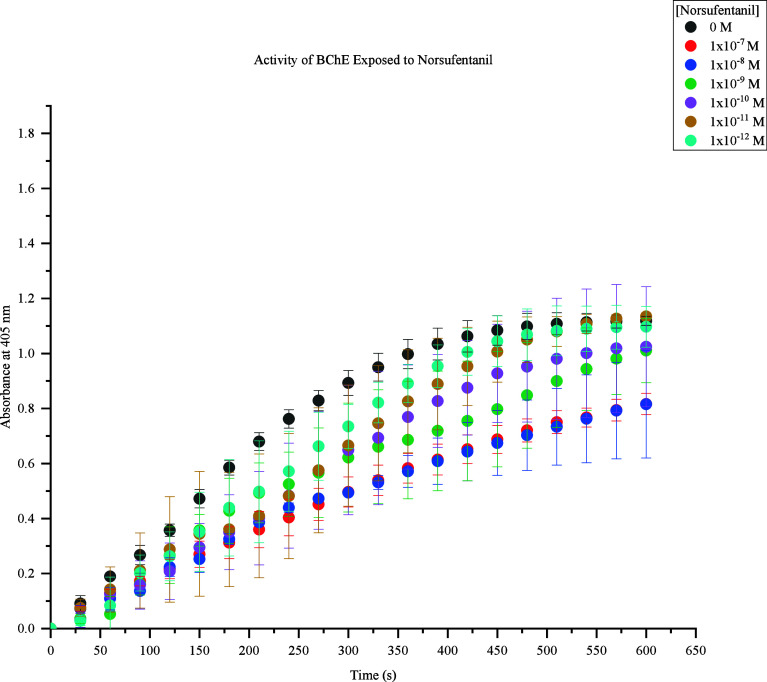
Activity of BChE Exposed to Norsufentanil. Response values at 405 nm range from approximately 0.8–1.1 at 600s. The highest concentration (1 × 10^–7^ M) produced the lowest response (0.8), and the lowest concentrations (1 × 10^–8^ M to 1 × 10^–12^ M) produced the highest responses (1.0–1.1). At 600s, 1 × 10^–8^ M to 1 × 10^–12^ M caused a decrease in BChE activity by 0.0–0.1 compared to the control samples.

The fifth fentanyl analogue assessed was norsufentanil. This FRS demonstrated insignificant and indistinguishable inhibition of BChE activity between 1 × 10^–11^ M and 1 × 10^–12^ M after 400s shown in Figure 6. Significant BChE inhibition was further expressed as the concentrations increased. Norsufentanil levels at 1 × 10^–7^ M and 1 × 10^–8^ M produced indistinguishable response values as well, which suggests norsufentanil does not exhibit differential effects on BChE activity between these values similar to 1 × 10^–11^ M and 1 × 10^–12^ M.

**Figure 7:**
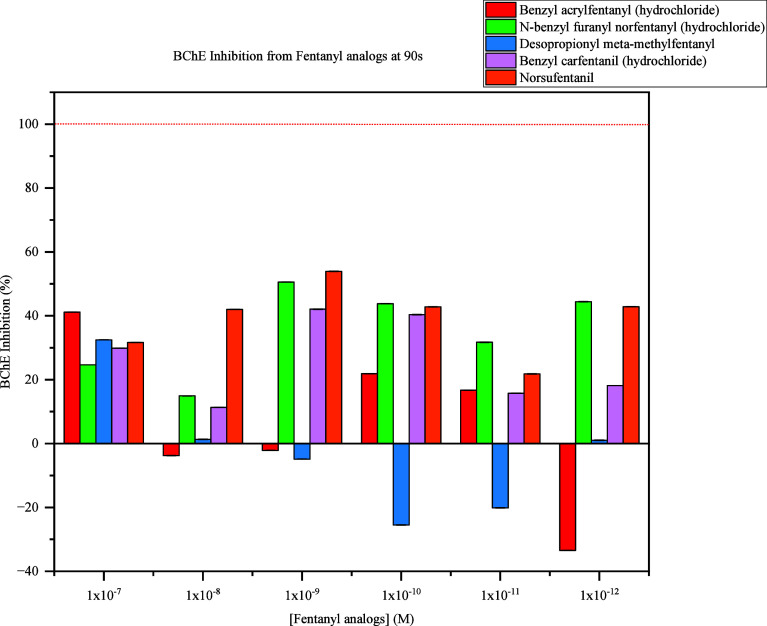
BChE Inhibition from all Fentanyl analogues at 90s. BChE inhibition expressed in percentages derived from control sample comparison when exposed to 1 × 10^–7^ M to 1 × 10^–12^ M levels of respective fentanyl analogues at 90s.

The primary aspects to consider for the evaluation of FRS-induced inhibition of BChE is the sensitivity and speed of the modified Ellman’s assay system. Therefore, BChE activity was evaluated at 90 and 600s. Figure 7 demonstrates the inhibition of BChE activity at all concentrations of each fentanyl analogue tested at 90s. The highest inhibition of BChE activity was observed with norsufentanyl at 41%. Negative percentages are caused by instrumentation background at the beginning of the analysis, but all fentanyl analogues exhibited inhibition within this time frame. When BChE was exposed to the highest concentration, 25%–41% inhibition was observed.

**Figure 8:**
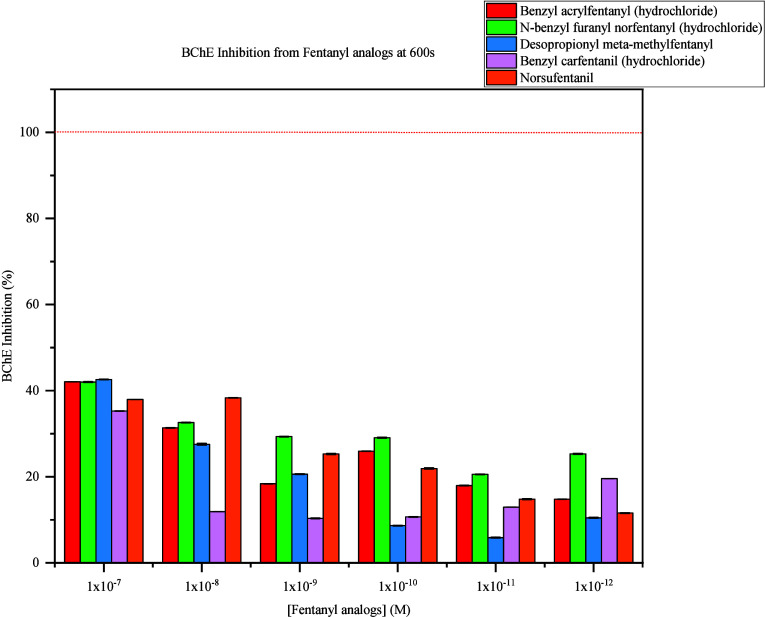
BChE Inhibition from Exposure to Fentanyl analogues. BChE inhibition is expressed in percentages derived from control sample comparison when exposed to all levels of respective fentanyl analogues at 600s. The highest inhibitory pattern correlates to the highest concentration (1 × 10^–7^ M), and the lowest inhibition correlates to the lowest concentrations (1 × 10^–12^ M) of fentanyl analogues tested.

The inhibition pattern of BChE from each fentanyl analogue at 600s is shown in Figure 8. The gradual decrease in BChE activity is observed from exposure to all 5 fentanyl analogues as concentrations increased. Each FRS demonstrated the highest BChE inhibition at 1 × 10^–7^ M which resulted in 43%–36% inhibition. Exposure to FRS at 1 × 10^–8^ M resulted in BChE inhibition of 38%–12%. At 1 × 10^–9^ M, FRS demonstrated BChE inhibition between 10%–29% while 1 × 10^–10^ M caused 29% to 8% BChE inhibition. Lastly, 1 × 10^–11^ M and 1 × 10^–12^ M demonstrated the lowest inhibition from 25%–6%.

**Scheme 1:**
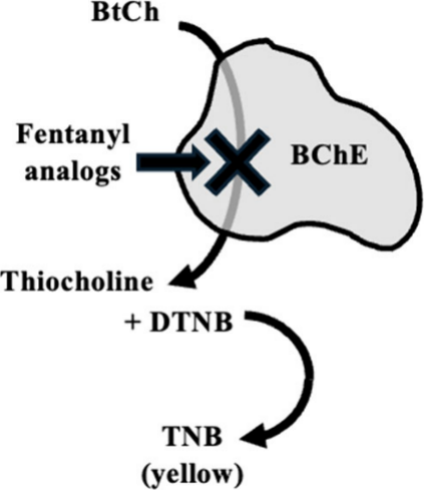
Addition of Fentanyl Analogues to Ellman’s Assay The fentanyl analogues interact with BChE and lower this reaction rate.

In summary, the availability of BChE to hydrolyze BtCh decreased when exposed to all fentanyl analogues compared to triplicates not exposed to these FRS. Thus, a decreased rate of BtCh hydrolysis by BChE resulted in lower concentrations of butyrate and thiocholine, which decreased the availability of interactions between DTNB and the thiol group in thiocholine. This was directly observable from the decreased color intensity development measured at 405 nm. Overall concentration dependence was observed, which is highlighted in the BChE inhibitory patterns from Figure 8. As the concentrations of FRS increased, BChE inhibition also increased and resulted in lower responses from the color intensity depicted in Scheme 1.

## Discussion

Considering the sensitivity and speed of the developed concept, all fentanyl analogues were evaluated at 90s and 600s. At the 90s interval, each fentanyl analogue caused significant BChE inhibition at 1 × 10^–7^ M. At 600s, BChE inhibition was observed with all fentanyl analogues at 1 × 10^–7^ M to 1 × 10^–12^ M with overall concentration dependence. Given various levels of BChE inhibition was observed, selectivity preventing detection of all FRS may cause current FTS to provide false negative results. A previous study revealed that the potency of fentanyl analogues varies based on molecular variations at para, ortho, and meta positions.^23^ These results confirm this concept reflected in the data presented as specific levels of BChE inhibition occurred when exposed to different fentanyl analogues, which is highlighted in the inhibitory patterns represented in Figure 8.

For example, it was discovered that benzyl carfentanil (hydrochloride) inhibited BChE activity at 30% during the 90s interval, but benzyl acrylfentanyl (hydrochloride) caused BChE inhibition at 41% during the 90s interval, which indicates the sensitivity of Ellman’s assay, but also introduces a nonselective method based solely on BChE inhibition for detection of FRS. However, analysis of these analogues at 600s provided increased distinguishability of inhibitory patterns observed for all FRS concentrations indicating the potential for more consistent and reliable detection of fentanyl analogues within 600s, but it does not hinder the ability of the developed concept to detect any fentanyl analogue within 90s.

## Conclusion

New developments provide a further understanding of fentanyl toxicity that can be used for robust detection of these extremely toxic compounds in field conditions. Results demonstrate significant inhibition of BChE when exposed to all fentanyl analogues and indicate the potential to utilize BChE activity as a basis for detecting FRS at 1 × 10^–7^ M within 90s, which highlights the sensitivity and efficiency of the developed method. The potential for a portable single-use kit to be developed for law enforcement and first responders would greatly impact the safety of public health nationwide as fentanyl can be detected from any surface at ng levels in less than 2 min with this method. This research also suggests that BChE inhibition levels can provide distinguishability between other illicit substances based on enzyme specificity. This kit can be utilized in any environment that allows confirmatory testing without an uncompromised speed and accuracy. While this method does not solve the problem of fentanyl exposure in the U.S., it can directly prevent overdoses from accidental exposure.
